# Aerobic exercise elevates perceived appetite but does not modify energy intake over a 3‐day postexercise period: A pilot study

**DOI:** 10.14814/phy2.70066

**Published:** 2024-09-27

**Authors:** Tetsuro E. Okada, Stewart Jeromson, Scott Rathwell, David C. Wright, Marc R. Bomhof

**Affiliations:** ^1^ Department of Kinesiology and Physical Education University of Lethbridge Lethbridge Alberta Canada; ^2^ School of Kinesiology University of British Columbia Vancouver British Columbia Canada; ^3^ Faculty of Land and Food Systems University of British Columbia Vancouver British Columbia Canada; ^4^ BC Children's Hospital Research Institute Vancouver British Columbia Canada

**Keywords:** aerobic exercise, appetite‐regulating hormones, energy compensation, energy intake, meal replacement beverage, perceived appetite

## Abstract

While a low degree of energy compensation is typically reported over the 24 h following a session of exercise, the prolonged impact of a bout of exercise on energy intake remains unclear. To overcome the challenge associated with accurately measuring energy intake in a free‐living environment, this study employed the use of a meal replacement beverage to assess the 3 day impact of an exercise session on energy intake. In a randomized, crossover study, 14 participants (8 male, 6 female) completed two trials: (1) EX: 75 min exercise on a motorized treadmill (75% VO_2peak_); and (2) SED: 75 min sedentary control session. Each condition was followed by 3 days of exclusive ad libitum consumption of a meal replacement beverage. Appetite‐regulating hormones, subjective appetite, energy intake, and energy expenditure were assessed. Exercise transiently suppressed the orexigenic hormone acyl‐ghrelin (*p* < 0.05) and elevated the appetite‐supressing hepatokine GDF‐15 (*p* < 0.05). Despite these acute changes, overall perceived appetite was elevated over the 3 day assessment period with exercise (*p* < 0.05). No increase in energy intake or change in postexercise physical activity patterns were observed. One acute session of moderate to vigorous exercise is unlikely to affect short‐term, three‐day energy balance in healthy individuals.

## 1 INTRODUCTION

Aerobic exercise is an effective method to maintain metabolic health and is often used as a strategy to control body weight (King, Hopkins, et al., 2009; Ross et al., 2020; Thomas et al., 2015; Warburton & Bredin, 2017). Despite the belief that exercise helps to support weight loss, habitual exercise is associated with a degree of energy compensation that can limit the amount of weight change achieved through exercise training (Flack et al., 2018; King et al., 2008; Melanson et al., 2013; Pontzer, 2022; Riou et al., 2015; Thomas et al., 2012). Energy compensation refers to the metabolic and behavioral adaptations that occur in response to exercise‐induced energy expenditure (EE) in order to maintain body energy stores (Doucet et al., 2018). One of the primary factors contributing to energy compensation is hunger and increased energy intake (EI) (Myers et al., 2019; Riou et al., 2015). The mechanisms and timeline associated with the energy compensation, however, remain unclear.

Acute exercise is widely purported to transiently suppress appetite (Dorling et al., 2018). The mechanisms mediating this response are related to changes in gut‐derived, appetite‐regulating hormones (Hazell et al., 2016). An acute bout of exercise has been shown to increase secretion of satiety hormones glucagon‐like peptide‐1 (GLP‐1) and peptide tyrosine tyrosine (PYY), while simultaneously suppressing the orexigenic hormone acyl‐ghrelin (Thackray & Stensel, 2023). In addition, new research demonstrates that exercise induces the release of the “heptokine” growth differentiation factor‐15 (GDF15). GDF15 increases in response to cellular and energetic stress, and when dosed pharmacologically has been shown to reduce food intake in rodents (Klein et al., 2021, 2022; Kleinert et al., 2018; Patel et al., 2019; Plomgaard et al., 2022). During the postexercise period, perceived ratings of appetite are generally reported to be lower (Howe et al., 2016; Thackray & Stensel, 2023). When participants are provided with an ad libitum postexercise meal, it is observed that relative EI, which subtracts the energy cost of exercise from EI, is reduced with exercise, generating a short‐term energy deficit (Donnelly et al., 2014; Riou et al., 2015; Schubert et al., 2013).

The reputed anorectic effects of exercise are in contrast to the longer‐term exercise interventions which show a considerable degree of energy compensation nearing or exceeding all additional EE from exercise (Myers et al., 2019; Riou et al., 2015, 2019). Long‐term exercise interventions report elevations in hunger and increased ad libitum intake with a meal challenge (Myers et al., 2019). The timeline associated with the transition from reduced relative EI to a period of energy compensation is not well understood. The majority of studies showing reductions in relative EI with exercise have been completed within a laboratory setting and are primarily focussed on the 8–24 h postexercise window (Balaguera‐Cortes et al., 2011; Deighton et al., 2013; Deighton & Stensel, 2014; Douglas et al., 2017). Beyond the 24 h period, energy compensation measures rely more heavily on participant‐completed food records, which are prone to random and systematic error, making it very difficult to accurately measure energy compensation (Casey et al., 2023; Poslusna et al., 2009; Stubbs et al., 2014). Furthermore, the studies that utilize ad libitum buffet meals or take‐home snack bags (Beaulieu et al., 2015; Douglas et al., 2015; King et al., 2015) may be affected by the “banquet effect,” whereby participants consume a greater amount of energy than under normal eating conditions (Beaulieu et al., 2015).

To overcome the challenge of accurately measuring EI in a free‐living environment beyond the 24 h postexercise window, we aimed to assess postexercise energy compensation using exclusive consumption of a standardized meal replacement beverage (MRB). To our knowledge, MRBs have not been previously utilized to assess postexercise EI. MRBs provide all essential nutrients and contain a set number of calories, making it relatively simple to calculate EI within a free‐living environment over an extended period. MRBs as a sole source of nutrition are well tolerated and have been successfully utilized in clinical settings (Heymsfield, 2010; Yolsuriyanwong et al., 2019). The overall objective of our study was to assess the prolonged effect of a single session of aerobic exercise on EI, subjective levels of appetite, appetite‐related hormones, and energy compensation in healthy, weight‐stable males and females over a 3‐day postexercise period.

## 2 MATERIALS AND METHODS

### 2.1 Participants

Fourteen participants (8 male; 6 female) were recruited from the University of Lethbridge and surrounding area. Participants were healthy, not actively attempting to lose or gain weight, and self‐reported weight stable. Exclusion criteria included taking medications that influenced appetite, smoking, initiation of a new oral contraceptive within the previous 3 months, and restrictive eating behavior. Participants provided written informed consent prior to taking part in the study. The study was approved by the University of Lethbridge Human Participant Research Committee (Ethics ID #2020–058) and was conducted in accordance with the ethical principles of the *Declaration of Helsinki*.

### 2.2 Preliminary session and test trial

Prior to baseline testing, participants reported to the lab for an initial screening session. Participants completed the Get Active Questionnaire (GAQ) to screen for contraindications to the exercise protocol (CSEP, 2017). Restrained eating was assessed using the Three‐Factor Eating Questionnaire Revised 18‐item (TFEQ‐R18) (de Lauzon et al., 2004; Karlsson et al., 2000). Individuals that scored ≥18 on the restrained eating subscale were classified as restrained eaters and were excluded from the study (Martins et al., 2010). The Godin's leisure time exercise questionnaire (GLTEQ) was used to assess recreational physical activity (Godin & Shephard, 1985). Following the initial screening session, participants were asked to complete a test trial to assess tolerance to a liquid diet to reduce potential dropout from the study. For the test trial, 6 bottles of a liquid MRB beverage (Ensure®Plus, Abbott Laboratories, Chicago, IL, USA) were provided to participants in a variety of flavors (chocolate, vanilla, and strawberry). With the exception of water, participants were asked to exclusively consume the provided MRB, starting with the first meal of the day and continue ad libitum consumption until the end of the day, or until the MRB drinks were all consumed. Participants that were able to tolerate the test trial and willing to participate in the study transitioned to baseline testing.

### 2.3 Baseline testing

Participant height and weight were recorded to the nearest 0.1 cm and 0.1 kg, respectively, on a calibrated Health‐o‐meter® professional weight scale (Pelstar® LLC, McCook, IL, USA). VO_2peak_ (mL O_2_·kg^−1^·min^−1^) was assessed using an incremental ramp test on a motorized treadmill, based on the modified Astrand protocol (Ferguson et al., 1998; Miller et al., 2007). Oxygen consumption and respiratory exchange ratio were continuously measured through breath‐by‐breath analysis using indirect calorimetry (COSMED, Concord, CA, USA). Heart rate was monitored via a Garmin heart rate monitor (HRM‐Dual, Garmin, Olathe, KS, USA). VO_2peak_ was determined to be the highest 30s rolling average across the test.

### 2.4 Experimental sessions

**FIGURE 1:**
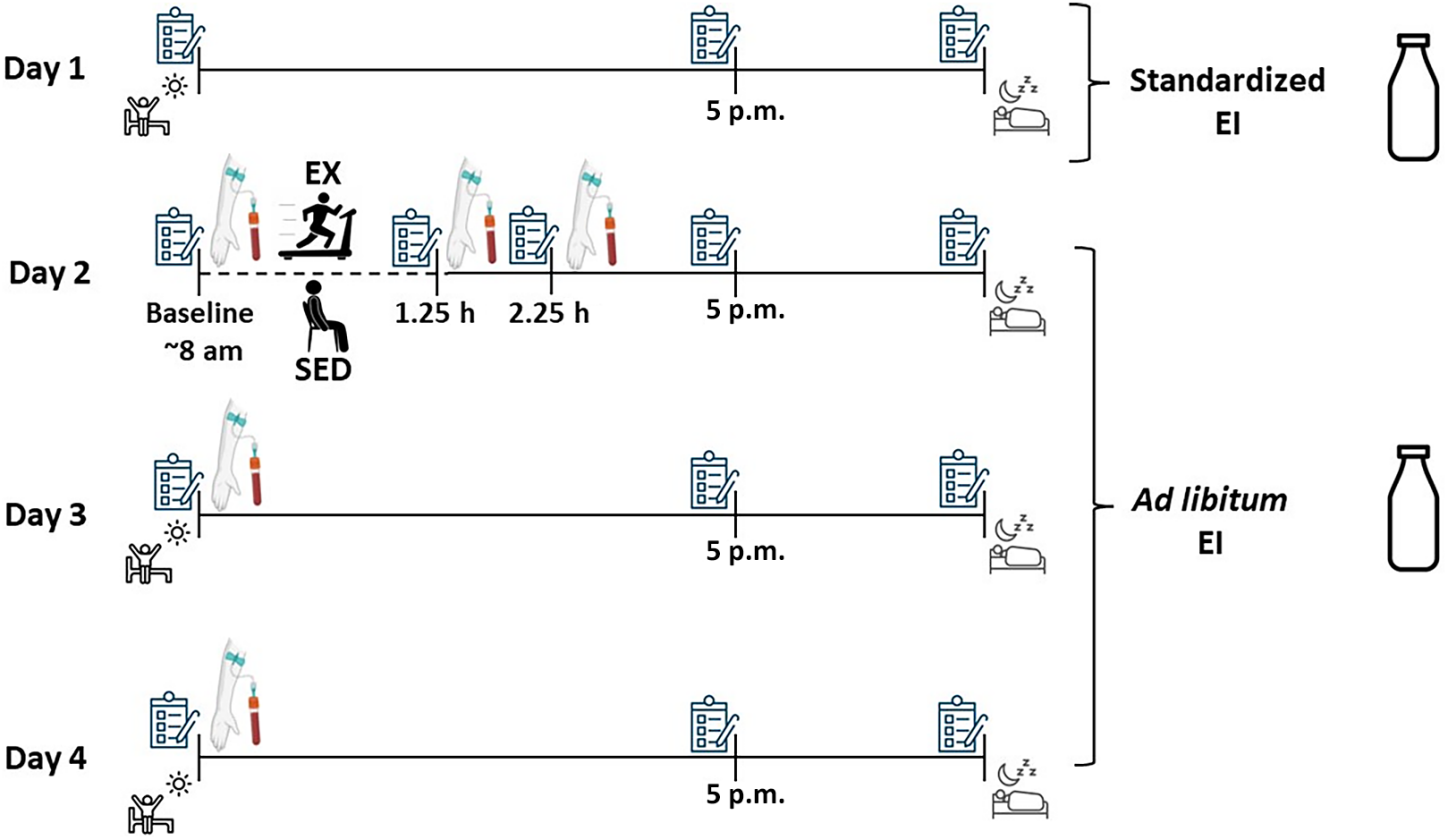
Timeline of experimental conditions. Treadmill/Sitting‐75 min of exercise (EX) or sedentary (SED) condition. Chart represents a visual analogue scale to record subjective measures of appetite. EI, Energy Intake.

Participants completed two, 4‐day crossover trials: (1) EX: Exercise (75 min of aerobic exercise performed at 75% VO_2peak_ on a motorized treadmill) and (2) SED: Sedentary control (75 min sedentary activity) in a randomized, counterbalanced order (Figure 1). Trials were scheduled a minimum 1 week apart for males and 4 weeks apart for females, within the early follicular phase of the menstrual cycle (day 1–10), to control for potential appetite fluctuations (Brennan et al., 2009; Campolier et al., 2016; Tucker et al., 2024; Van Vugt, 2010). For each trial, participants were required to exclusively consume the provided MRBs (Ensure®Plus, Abbott Laboratories, Chicago, IL, USA) and one daily fiber bar (NuGo Fiber d'Lish, NuGo Nutrition, Oakmont, PA, USA). Each bottle of MRB provided 350 kcal (1.5 kcal/mL fluid); 11 g fat; 48 g carbohydrate (1 g fiber); and 16 g protein. All labels on the MRBs were removed. The fiber bar provided 150 kcal, 3 g fat, 31 g carbohydrate (8 g soluble fiber, 4 g insoluble fiber), and 3 g protein.

Prior to each session, participants were asked to refrain from strenuous exercise, alcohol, and caffeinated beverages in the 24 h leading into the trial. On day 1 of the first randomized trial, participants were provided with surplus MRB and asked to consume the drinks ad libitum. The number of bottles of MRB initially provided to each participant was set at 2× the daily energy requirements, based on the Harris‐Benedict formula and activity factors. To standardize EI on day 1 of the 2nd trial, participants were provided with the same amount of energy they consumed on day 1 of the first trial. On day 2, participants reported to the lab at ~0800 h following an overnight fast (>10 h). Fasting was verbally confirmed in the morning. After a fasting blood collection via venipuncture, participants completed the 75 min session of sedentary control or 75 min of aerobic exercise at 75% VO_2peak_. Within the *heavy* intensity exercise domain for most individuals, a 75% VO_2peak_ for 75 min challenges physiological homeostasis, places high demand on glycogen as a fuel source, and is considered to be “uncomfortably sustainable” for participants (Iannetta et al., 2019; Keir et al., 2022). To verify exercise intensity at 75% VO_2peak_ during the exercise sessions, participants held the metabolic cart mask to their face for ~1 min at 30 min and 60 min during the exercise sessions. Additional blood samples via venipuncture were collected immediately following the cessation of exercise/sedentary session and 1 h post. After completing the exercise or sedentary condition (~135 min) participants were free to leave the laboratory and begin ad libitum intake of MRB and water. For days 2 to 4 of each trial, participants were provided a surplus of MRB to ensure that participants had an adequate supply of energy for each day. The number of bottles of MRB provided on a daily basis was set at the bottles required to meet 3× basal metabolic rate (determined using the Harris‐Benedict formula). On days 3 and 4 of each trial, participants reported to the laboratory in the morning at ~0800 h following an overnight fast to return all empty, partially empty, and full bottles of MRB to confirm EI. Adherence to the exclusive consumption of the provided MRBs and fiber bars was verbally confirmed. MRBs and fiber bars were restocked on day 3 and 4. On average, participants received 14 bottles on MRB daily (4900 kcal). Throughout each trial, participants were permitted to consume plain tea and coffee. Coffee and tea intake were recorded by participants during the first trial and matched for the second trial.

Participants completed a visual analogue scale (VAS) to assess subjective measures of hunger, satisfaction, prospective food consumption (PFC), and fullness (Flint et al., 2000). Perceived appetite was assessed on days 1–4 of both trials when participants woke from sleep, around dinner time (~1700 h), and just prior to sleep. Additional appetite recordings were completed on day 2 of each trial immediately after and 1 h following the exercise and sedentary sessions. A composite satiety score was calculated using the formula: CSS (mm) = (satisfaction + fullness + (100−PFC) + (100−hunger))/4 (Gilbert et al., 2012).

### 2.5 Measurement of activity level

Baseline levels of physical activity were measured using the GLTEQ (Godin & Shephard, 1985). An overall weekly mean Metabolic Equivalent of Task (MET)·hours was determined by multiplying the number of weekly hours each participant spent in mild (e.g., easy walking), moderate (e.g., fast walking), and vigorous physical activity (e.g., running) by an estimated value of 3 MET·h, 5 MET·h, and 9 MET·h, respectively.

Free‐living activity (daily sleeping, lying, sitting, standing and ambulating, step counts, and MET values) throughout each 4‐day trial was measured with an activPAL4™ (PAL Technologies Ltd., Scotland, UK) inclinometer. The device was secured to the quadriceps of each participant using medical tape. To reduce skin irritation, the activPAL4™ device was wrapped in a finger cot. Daily MET values were multiplied by the estimated basal metabolic rate using the Harris‐Benedict equation to estimate EE (Duvivier et al., 2013). Daily EE estimations with the activPAL have been measured to be within 15% of EE determination using a portable gas analyzer (Alberto et al., 2017). Energy balance was determined by subtracting total EE from daily EI (Energy balance = total EI – total energy expenditure).

### 2.6 Biological analysis of satiety hormones

All blood samples were collected into pre cooled 6 mL K_2_Ethylenediaminetetraacetic acid spray‐coated vacutainers (BD, Mississauga, ON, Canada). Immediately after collection, a protease inhibitor cocktail containing dipeptidyl peptidase IV inhibitor (10 μL/mL blood; #DPP4‐M, MilliporeSigma Corp. Burlington, MA, USA), sigma protease inhibitor (1 mg/mL blood; #S8820, SigmaFast, MilliporeSigma Corp. Burlington, MA, USA), and pefabloc (1 mg/mL blood; #76307, MilliporeSigma Corp. Burlington, MA, USA) was added to the sample to prevent degradation of appetite‐related hormones. Blood samples were centrifuged at 2500 *g* for 10‐min at 4°C. Plasma aliquots were stored at −80°C for later analysis. The concentration of PYY was determined using the Human PYY (Total) ELISA kit (#EZHPYYT66K, MilliporeSigma Corp. Burlington, MA, USA). Circulating GLP‐1 concentrations were assessed using the High Sensitivity GLP‐1 Active Chemiluminescent ELISA kit (#EZGLPHS‐35 K, MilliporeSigma Corp. Burlington, MA, USA). Acyl‐ghrelin was assessed by the Human Ghrelin (Active) kit (#EZGRA‐88 K, MilliporeSigma Corp. Burlington, MA, USA). GDF‐15 levels were measured as previously described using a commercially available ELISA assay (#SGD150, R&D systems, Minneapolis, MN, USA) (Plomgaard et al., 2022). All samples were assayed in duplicate. The intra‐ and inter‐assay variation for these assays ranged between 2% and 7% in our laboratory, as previously described (Hamilton et al., 2020).

### 2.7 Statistical analysis

SPSS software v28.0 for Windows was used to analyze the data. Our primary outcome measure was EI between the exercise and sedentary trials. Sample size estimations were completed using G*Power (*α* = 5%, *β* = 80%) using an effect size of 0.7 and the analysis of variance (ANOVA): repeated measures, within factors statistical test. The effect size was based on a previously conducted pilot study conducted in our lab. Data was assessed for normality using the Shapiro–Wilk test. Differences in acyl‐ghrelin, active GLP‐1, total PYY, GDF‐15, subjective appetite, EI, EE, and energy balance were analyzed using a two‐way, repeated measures ANOVA. If a significant condition × time interaction was observed, pairwise comparisons were used to determine between condition differences. Within‐condition differences were examined using a one‐way, repeated measures ANOVA followed by a post hoc Bonferroni analysis. Effect size for repeated measures ANOVA was determined using partial eta squared (*η* ^2^), while effect size for post hoc pairwise comparisons was calculated using Cohen's *d*. The magnitude of effect size was determined by the following criteria: small (*η* ^2^ = 0.01; *d* = 0.2), medium (*η* ^2^ = 0.06; *d* = 0.5), and large (*η* ^2^ = 0.14; *d* = 0.8). Correlation was assessed using a Pearson correlation test. AUC estimations were calculated using trapezoidal sums and analyzed using pairwise comparisons. Statistical significance was set at *p* < 0.05. All data is represented as mean ± standard deviation (SD).

## 3 RESULTS

### 3.1 Participants and baseline measurements

A total of 25 participants were initially recruited for the study. Two participants were excluded from the study due to restrained eating (TFEQ‐R18 score ≥ 18). Twenty‐three participants completed the test trial phase of the study, with 9 participants reporting that they could not tolerate an exclusive MRB diet for 4 consecutive days. Of the 14 participants that initiated the full study, all 14 completed both trials. Participant characteristics are highlighted in Table 1. Participants had a mean restrained eating score of 13.0 ± 3.1. Female participants began the exercise and sedentary trial on day 5.0 ± 1.4 and 6.0 ± 1.4, respectively, of the menstrual cycle.

**TABLE 1:** Participant Characteristics.

Mean ± SD (range)			
	Total (*n* = 14)	Females (*n* = 6)	Males (*n* = 8)
Age (years)	23.6 ± 3.4 (18–31)	23.0 ± 3.5 (18–27)	24.0 ± 3.5 (20–31)
Height (m)	1.7 ± 0.1 (1.6–1.8)	1.7 ± 0.1 (1.6–1.8)	1.8 ± 0.1 (1.6–1.8)
Weight (kg)	73.8 ± 14.2 (50.8–98.8)	62.7 ± 9.8 (50.8–78.2)	82.2 ± 11.0 (67.0–98.8)
BMI (kg·m^−2^)	24.7 ± 3.6 (17.1–29.9)	22.7 ± 4.0 (17.1–28.7)	26.3 ± 2.5 (22.3–30.0)
WC (cm)	83.5 ± 7.6 (71.7–96.0)	78.8 ± 6.9 (71.7–90.1)	87.1 ± 6.4 (77.7–96.0)
MET·h·wk^−1^	43.9 ± 30.1 (3.0–89.0)	43.3 ± 26.3 (6.8–86.0)	44.8 ± 33.8 (3.0–89.0)
VO_2peak_ (mL O_2_·kg^−1^·min^−1^)	41.5 ± 4.8 (35.4–50.5)	41.0 ± 6.2 (35.4–50.5)	41.8 ± 3.9 (36.0–45.7)

Abbreviations: BMI, Body mass index; MET, Metabolic equivalent of task; WC, Waist circumference.

### 3.2 Appetite‐regulating hormones

#### 3.2.1 Acyl‐ghrelin

**FIGURE 2:**
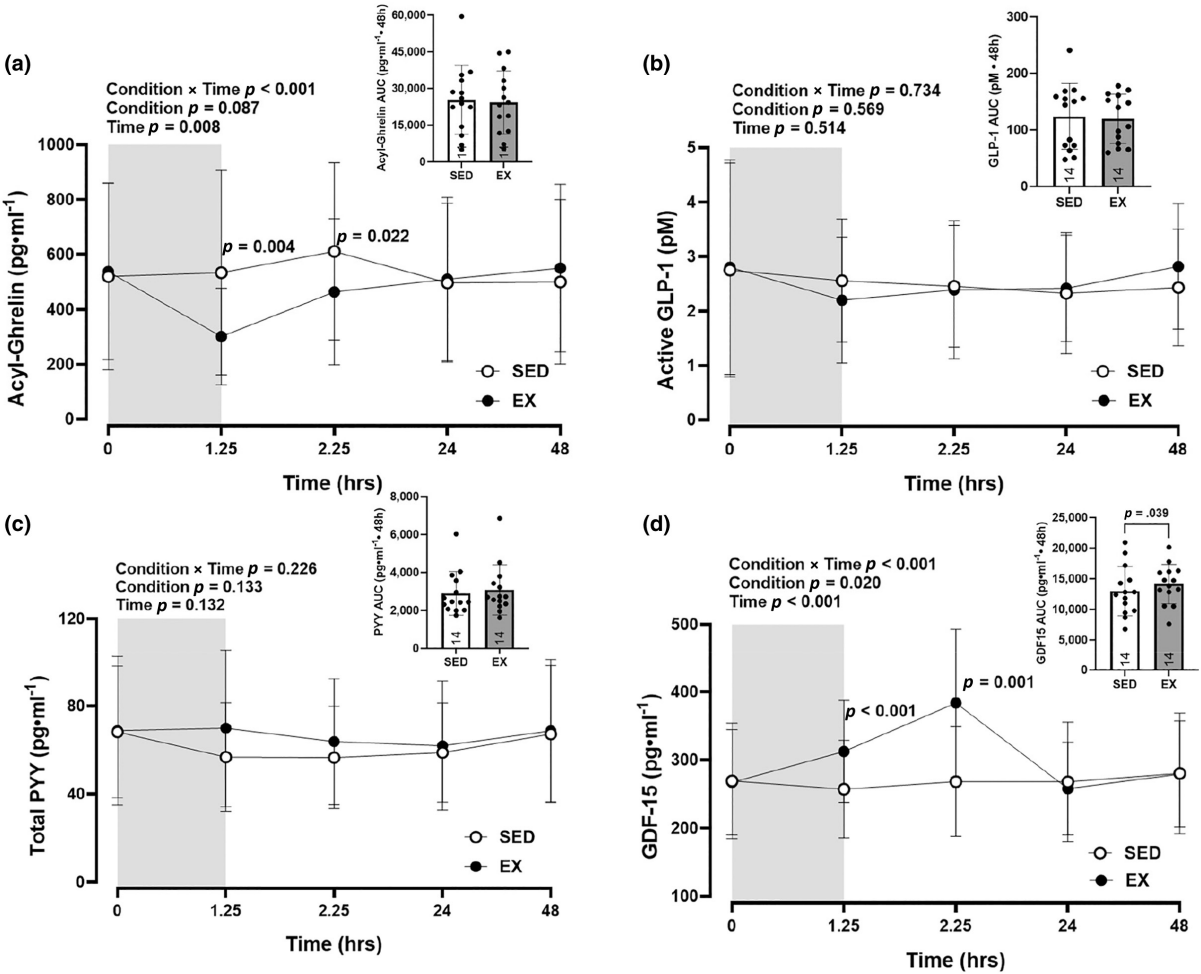
Appetite‐regulating hormones. Concentrations and area under the curve (AUC) for (a) Acyl‐ghrelin, (b) Active GLP‐1, (c) Total PYY, and (d) GDF‐15 between SED and EX condition. The gray shaded rectangles represent 75 min of EX or SED. Values are means ± SD, *n* = 14. EX, exercise; GDF‐15, growth differentiation factor‐15, GLP‐1, glucagon‐like peptide 1; PYY, peptide tyrosine tyrosine; SED, sedentary.

A condition × time interaction (*p* < 0.001) was observed for concentrations of acyl‐ghrelin (Figure 2a). *Post hoc* pairwise comparisons demonstrated lower levels of acyl‐ghrelin during the exercise condition immediately postexercise (*p* = 0.004; *d* = 0.92) and 60 min postexercise (*p* = 0.022; *d* = 0.69), compared to the sedentary condition. Within‐condition, fasted concentrations of acyl‐ghrelin were reduced immediately postexercise (*p* = 0.016) in EX. Within SED, levels of acyl‐ghrelin increased 60 min post sedentary session (*p* = 0.045) relative to baseline. Acyl‐ghrelin AUC was negatively correlated with total EE (*r* = −0.441, *p* = 0.019) and positively correlated with energy balance (*r* = 0.387, *p* = 0.042).

#### 3.2.2 Active GLP‐1

No condition × time interaction (*p* = 0.734), main effect of condition (*p* = 0.569; *η* ^2^ = 0.03), or main effect of time (*p* = 0.514) were observed for active GLP‐1 (Figure 2b).

#### 3.2.3 Total PYY

No condition × time interaction (*p* = 0.226), main effect of condition (*p* = 0.133; *η* ^2^ = 0.17), or main effect of time (*p* = 0.132) were observed for concentrations of total PYY (Figure 2c).

#### 3.2.4 GDF15

A condition × time interaction (*p* < 0.001) was observed for GDF15 (Figure 2d). *Post hoc* pairwise comparisons demonstrated greater levels of GDF‐15 during the exercise condition immediately (*p* < 0.001; *d* = 1.19) and 60 min postexercise (*p* = 0.001; *d* = 1.47), compared to the sedentary condition. Within‐condition, concentrations of GDF‐15 were increased immediately (*p* = 0.028) and 60 min postexercise (*p* = 0.007). AUC was greater in EX relative to SED (*p* = 0.039). There was a negative correlation between GDF‐15 immediately after the EX/SED session and total EE (*r* = −0.416, *p* = 0.028). GDF‐15 AUC was positively correlated with CSS (*r* = 0.484, *p* = 0.009).

### 3.3 Perceived appetite

**FIGURE 3:**
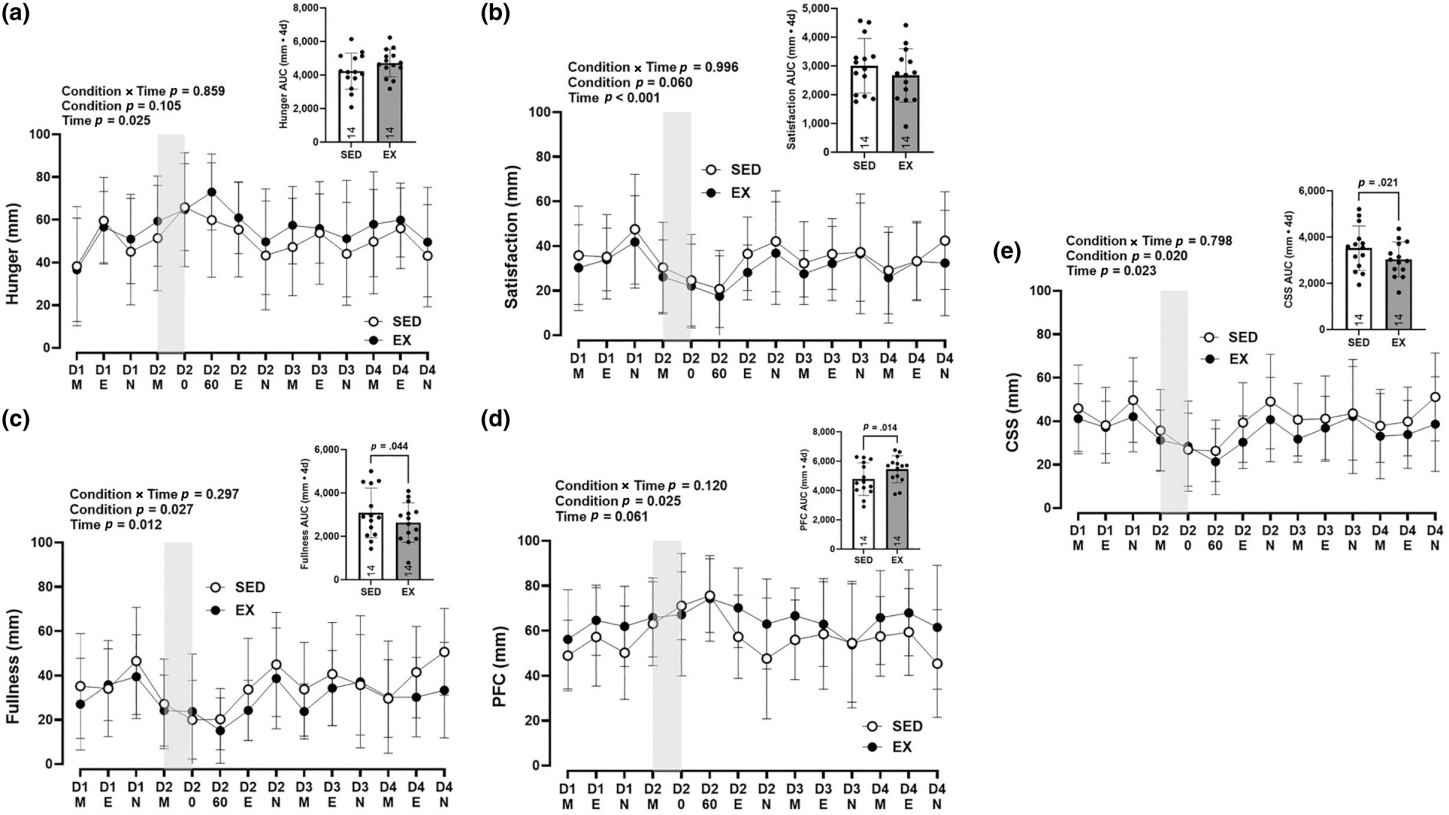
Subjective measures of appetite. Ratings and area under the curve (AUC) for perceived (a) Hunger, (b) Satisfaction, (c) Fullness, (d) PFC, and (e) Composite satiety score on a (*i*) 100 mm VAS [D*_*, day 1, 2, 3, or 4; M, morning; E, evening; N, night; 0, immediately post‐EX/SED; 60, 60‐min post‐EX/SED] between SED and EX conditions. Gray shaded rectangles represent 75 min of EX or SED. Values are means ± SD, *n* = 14. EX, exercise; PFC, prospective food consumption; SED, sedentary; VAS, visual analogue scale.

No condition × time interactions were observed for any measures of subjective appetite (hunger, *p* = 0.859; satisfaction, *p* = 0.996; fullness, *p* = 0.297; PFC, *p* = 0.120) (Figure 3). There was a main effect of time for perceived hunger (*p* = 0.025), satisfaction (*p* < 0.001), and fullness (*p* = 0.012), but not PFC (*p* = 0.061). A main effect of condition was observed for perceived fullness (*p* = 0.027; *η* ^2^ = 0.32) and PFC (*p* = 0.025; *η* ^2^ = 0.33), with participants feeling less full and able to eat more during the exercise condition. There was no main effect of condition for hunger (*p* = 0.105; *η* ^2^ = 0.19) or satisfaction (*p* = 0.060; *η* ^2^ = 0.25). AUC was lower for fullness (*p* = 0.044) and greater for PFC (*p* = 0.014) in EX relative to SED. There was a main effect of condition (*p* = 0.020; *η* ^2^ = 0.35) and time (*p* = 0.023) for CSS. AUC for CSS was lower in EX (*p* = 0.021), indicating and an overall lower level of satiety with exercise. CSS AUC was negatively correlated with total EE (*r* = −0.537, *p* = 0.003).

### 3.4 Activity tracking

**FIGURE 4:**
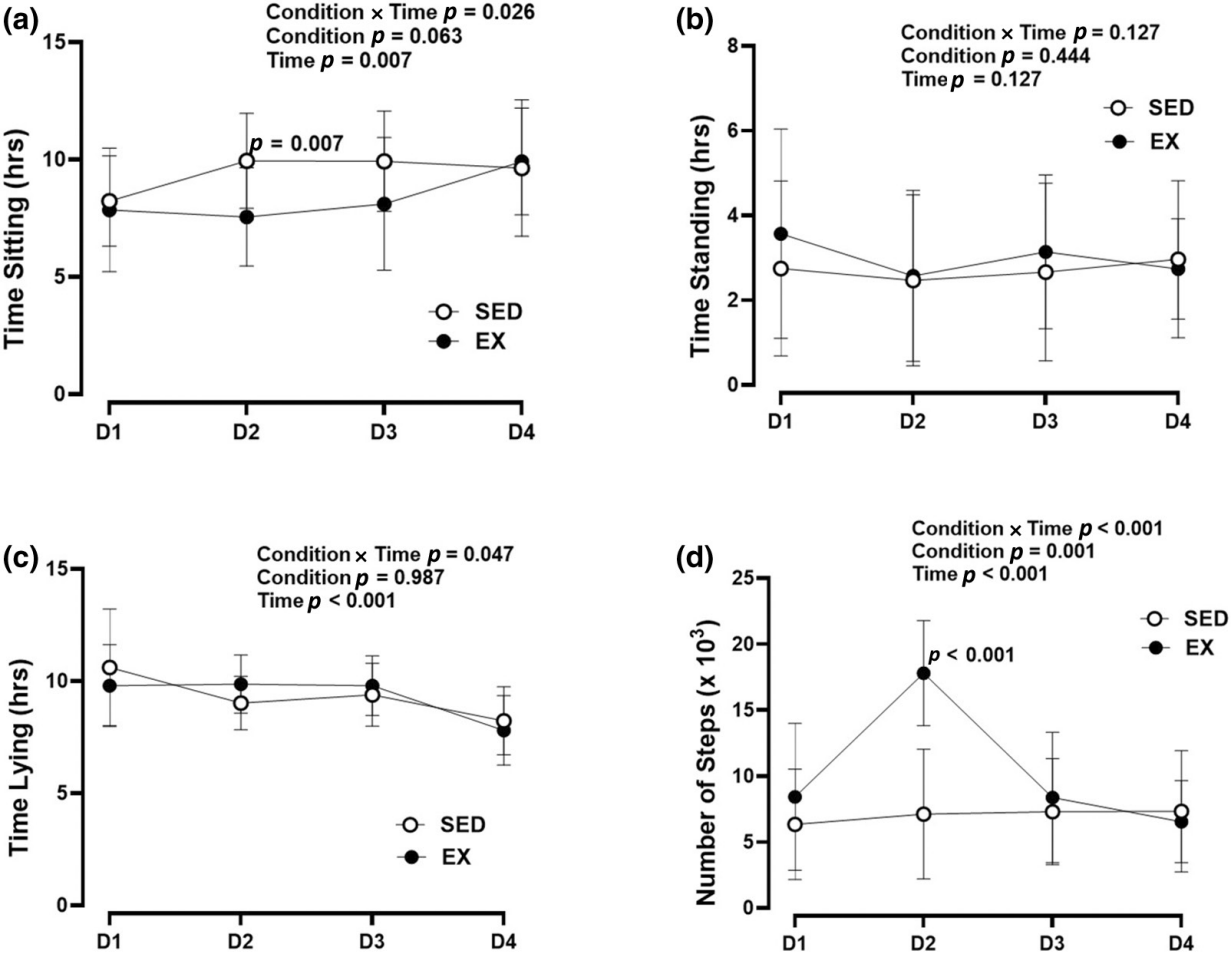
ActivPAL events. Recorded measures of (a) Time sitting, (b) Time standing, (c) Time lying, and (d) Number of steps using the ActivPal over a 4‐day time frame. EX, exercise; SED, sedentary.

A condition × time interaction effect was observed for time spent sitting (*p* = 0.026), lying (*p* = 0.047), and number of steps (*p* < 0.001) (Figure 4). Post hoc analysis demonstrated that more time was spent sitting on day 2 in the sedentary condition (*p* = 0.007; *d* = 0.86). Participants had higher step counts on day 2 (*p* < 0.001, *d* = 1.86) and total steps (*p* = 0.001, *d* = 1.18) during the exercise condition. There was a trend for more time seated on day 3 (*p* = 0.054; *d* = 0.57) and total time seated (*p* = 0.063; *d* = 0.54) during the sedentary condition compared to exercise.

### 3.5 Energy intake, energy expenditure, and energy balance

#### 3.5.1 Energy intake

**FIGURE 5:**
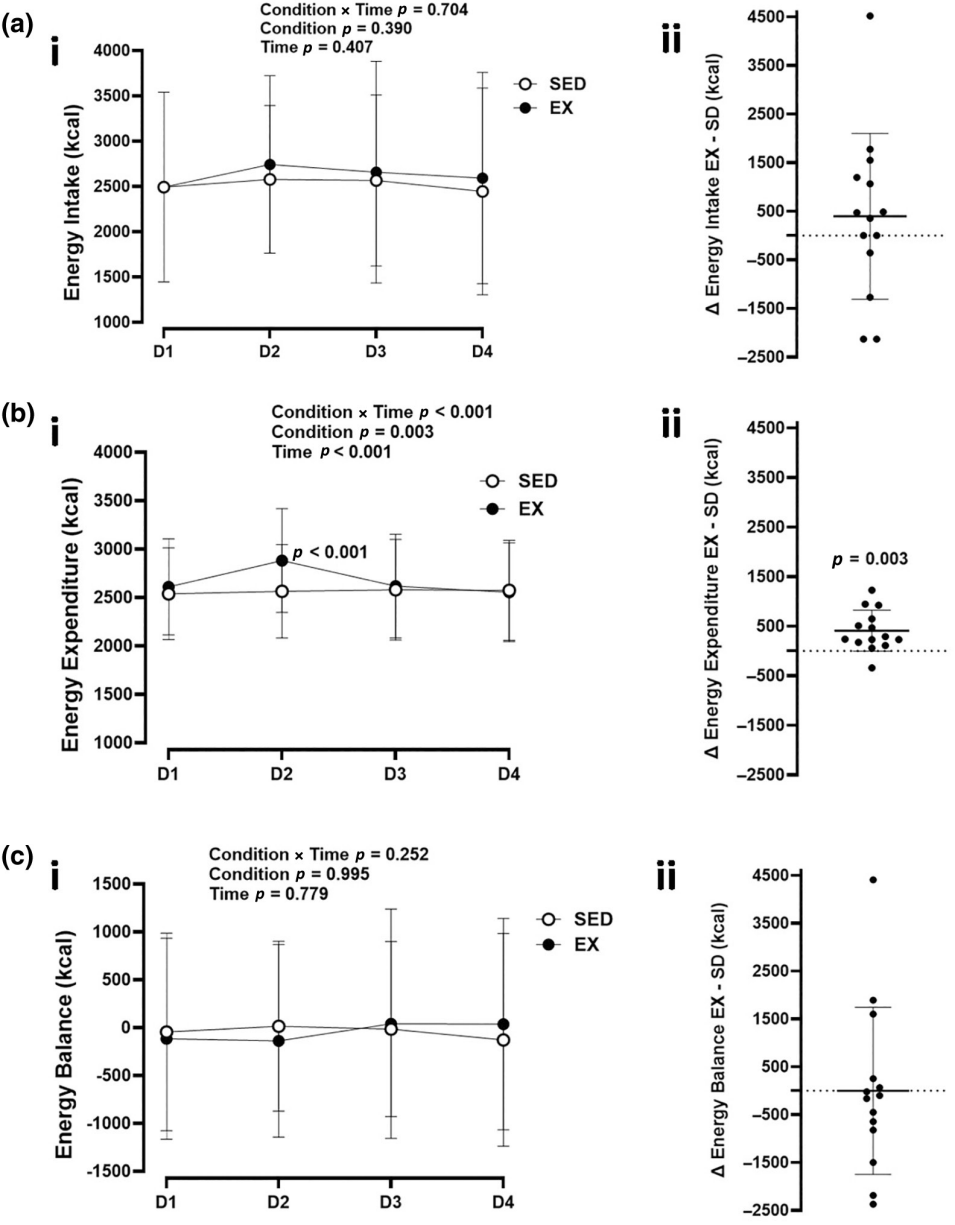
Daily and individual measures of energy intake, energy expenditure, and energy balance. (i) Daily and (ii) individual overall change in (a) Energy intake (EX condition−SD condition), (b) Energy expenditure; (c) Energy Balance over 4 days. Values are means ± SD, *n* = 14. EX, exercise; SED, sedentary.

There was no condition × time interaction (*p* = 0.704), main effect of condition (*p* = 0.390; *η* ^2^ = 0.06), or main effect of time (*p* = 0.407) for EI throughout the trials (Figure 5a). Total EI (*p* = 0.401; *d* = 0.23) was the same between the exercise and sedentary conditions.

#### 3.5.2 Energy expenditure

A significant condition × time interaction (*p* < 0.001) was observed for EE between the exercise and sedentary conditions (Figure 5b). Participants expended more energy on day 2 (*p* < 0.001; *d* = 1.74) and across the full, 4‐day trial (*p* = 0.003; *d* = 0.99) during the exercise condition compared to sedentary.

#### 3.5.3 Energy balance

No condition × time interaction (*p* = 0.252), main effect of condition (*p* = 0.995), or main effect of time (*p* = 0.779) were observed for energy balance between the exercise and sedentary conditions (Figure 5c). Daily (Day 1: *p* = 0.065, *d* = 0.54; Day 2: *p* = 0.427, *d* = 0.22; Day 3: *p* = 0.779, *d* = 0.08; Day 4: *p* = 0.302, *d* = 0.29) energy balance was the same between conditions.

## 4 DISCUSSION

This study investigated the impact of a single bout of aerobic exercise (75% VO_2peak_) on postexercise appetite, EI, and EE over a 3‐day postexercise period. Transient anorexigenic changes in appetite‐related hormones were observed with reduced acyl‐ghrelin and increased GDF15 immediately and 60 min postexercise. No prolonged, 3‐day postexercise differences were observed for appetite‐regulating hormones or measures of energy compensation. Despite these changes, participants felt less full and had a higher desire to eat during the exercise condition. This was further supported by a lower CSS, indicating reduced levels of perceived satiety with exercise. As expected, activity tracking demonstrated higher EE on the day of exercise, contributing to greater overall EE in the exercise condition, despite no difference in EE on day 3 and 4. Taking into account daily and total EI and EE, there were no overall differences in energy balance between the exercise and sedentary conditions.

A session of aerobic exercise at 75% VO_2peak_ typically elicits an acute suppression of subjective appetite, reduction in acyl‐ghrelin, and increase in GLP‐1 and PYY (Thackray & Stensel, 2023). Here we observed a transient suppression of acyl‐ghrelin immediately and 60 min postexercise. No changes were observed with active GLP‐1 and total PYY. The specific reason for the null result with GLP‐1 and PYY, despite seeing changes in acyl‐ghrelin, is not clear. Despite robust evidence that exercise elicits reductions in acyl‐ghrelin, many studies do not report changes in anorectic hormones GLP‐1 and PYY with acute exercise (McCarthy et al., 2024). With participants entering the exercise sessions in a fasted state, coupled with the prolonged period of aerobic exercise, it is possible that there was a blunting of the GLP‐1 and PYY response to exercise. Confirming what has been demonstrated in previous human studies, we found that exercise increased GDF15 levels by ~43% (Kleinert et al., 2018; Plomgaard et al., 2022). It remains unclear as to whether exercise‐induced changes in GDF15 are sufficient to reduce appetite in humans (Klein et al., 2021). Research conducted in a preclinical animal model suggests that endogenous increases in GDF15 achieved through exercise are not sufficient to suppress food intake; rather, it is only when levels are elevated through pharmacological means that EI is suppressed (Klein et al., 2021). Here we confirm that EI was not reduced on the day of exercise. Additionally, subjective appetite ratings did not differ between groups on day 2 of the trials. Together, this suggests that the changes in appetite‐regulating hormones were not sufficient to modify appetite or suppress EI. While it is common for EI to remain the same following a bout of exercise, relative EI is often reduced when accounting for the cost of the exercise session (Schubert et al., 2013). In our study, total EE on the day of exercise was greater than the sedentary condition. However, when looking at the difference between total daily EI and EE on day 2, there were no significant differences between conditions. This finding can be attributed to the high variability observed in EI measurements relative to EE. While EI was numerically greater in the exercise condition on day 2 (~166 kcal), the results were highly variable. Incorporating the variability from EI into the EB calculation (EI‐EX), the difference in EB was not significant, despite a numerical difference of ~151 kcal between the EX and SED conditions on day 2. Overall, while sufficient to induce aspects of the exercise‐induced anorectic response, no short‐term energy deficits were generated.

Few studies explore energy compensation extending beyond the 24 h postexercise period. On day 3 and 4 of the exercise trial, we observed no differences in fasting levels of active GLP‐1, total PYY, acyl‐Ghrelin, or GDF15. This pattern is similar to what has been demonstrated by three previous studies. With 2 × 60 min treadmill running sessions (70% VO_2peak_), Douglas et al. (2015) demonstrated that exercise had no influence on fasting levels of acyl‐ghrelin or PYY. Furthermore, a sprint interval session was found to have no impact on fasting levels of GLP‐1 and PYY (Beaulieu et al., 2015). King et al. (2015), in response to a 90 min treadmill session (70% VO_2peak_), observed no major differences in PYY and a slightly reduced level of acyl‐ghrelin on the day following exercise. Extending the findings of these studies by an additional 24 h, we observed that the levels of these hormones remained stable after an overnight fast up to 48 h postexercise. Long‐term exercise studies conducted in individuals with overweight have reported increases in fasting concentrations of PYY (Jones et al., 2009; Rosenkilde et al., 2013) after 3–8 months. In contrast, prolonged exercise interventions have also noted increases in acyl‐ghrelin and reductions in GLP‐1 (Flack et al., 2018; Ouerghi et al., 2021). The timeline and relative contribution of episodic hormones to energy compensation with exercise training remains unclear. Based on our study, it would appear that short‐term appetite hormones have a limited role in mediating postexercise appetite in the days following an exercise session. The potential role of episodic hormones in regulating long‐term appetite with exercise requires further investigation.

The short‐term studies that have examined EI for an extended period beyond 24 h postexercise have found no elevations in EI. Douglas and colleagues observed no changes in total EI over a 2‐day period with aerobic exercise (70% VO_2peak_) (Douglas et al., 2015). Furthermore, Beaulieu et al. (2015), comparing a session of sprint interval training to sedentary control, showed no differences in total EI over 34 h. In both of these studies, EI was assessed with ad libitum laboratory meals and take‐home snack bags. Participants in both the exercise and sedentary conditions were found to overconsume during the ad libitum meals, creating a large positive energy balance. This effect was speculated to be a result of a “banquet effect,” whereby participants overconsumed due to an abundance of palatable food items (Beaulieu et al., 2015; Douglas et al., 2015). Here, utilizing an MRB to assess EI, we show that EI was not different between the exercise and sedentary condition. Daily EI remained very similar to daily EE, suggesting that MRBs may not elicit the same degree of overconsumption with ad libitum meals. Extending the postexercise timeline beyond 3 days, 7–14 day exercise interventions incorporating regular sessions of exercise have found some degree of energy compensation through increased EI (Stubbs, Sepp, Hughes, Johnstone, King, et al., 2002; Whybrow et al., 2008). Based on the results of our study and others, it is likely that repeated exposure to exercise is necessary to induce compensatory changes in EI.

Although there were no differences in EI, perceived appetite was affected by exercise. A reduction in fullness, increase in PFC, and overall lowering of CSS was found in the exercise condition over the 4 day trial. The overall reduction in AUC for CSS was ~14%. On the evening of day 2 and the morning of day 3, the average CSS ratings differed by ~8 mm between EX and SED. Based on a previous research investigating the impact of exercise interventions on appetite, the differences observed in our study would be considered negligible and unlikely to have a discernable impact on eating behavior (Beaulieu et al., 2021). We observed that the AUC for CSS was negatively correlated with total EE, indicating that a greater EE was associated with a lower level of satiety. The finding of elevated appetite following exercise, even with no increase in EI, has been previously reported (Beaulieu et al., 2015). In response to sprint interval exercise, ratings of hunger and motivation to eat were heightened over a 2‐day period. Ratings of hunger have also been found to increase with a daily exercise session over a 1 week span in a group of females (Stubbs, Sepp, Hughes, Johnstone, King, et al., 2002). In short‐term studies, the explanation for elevated appetite in the absence of increased EI remains somewhat unclear. Based on a systematic review of prolonged exercise interventions, the elevation in perceived hunger with little to no increase in overall EI, may be due to increased dietary restraint and reduced disinhibition (Beaulieu et al., 2021). Evidence from King, Caudwell, et al. (2009) also purports that exercise may enhance the sensitivity of the appetite control system, resulting in a greater satiety response to a fixed volume of food. Thus, a session of exercise may elevate hunger, but due to improved sensitivity of the homeostatic appetite signaling system, increased dietary restraint, and decreased disinhibition, EI may not be driven higher by exercise.

Previous studies have demonstrated that a reduction in nonexercise activity thermogenesis (NEAT), rather than an increase in EI is responsible for energy compensation (Riou et al., 2015). We observed that overall EE was higher with exercise, primarily due to the increase in EE on day 2, with noted increases in the number of steps and reduced time sitting with exercise. On day 3 and 4, no differences were observed in EE or activity tracking between the two conditions. A similar result has been reported by Cadieux et al. (2014), with assessment of NEAT over a 34 h window. Longer‐term 3 month (Kozey‐Keadle et al., 2014; Myers et al., 2019), 8 month (Hollowell et al., 2009), and 10 month (Willis et al., 2014) exercise interventions have also reported that NEAT levels remain similar with exercise. There are, however, a number of studies that contrast these findings. Kriemler et al. (1999), in a group of adolescent males, observed reduced EE and spontaneous physical activity on the same day of moderate or strenuous exercise. Prolonged studies, over a 7 day (Stubbs, Sepp, Hughes, Johnstone, Horgan, et al., 2002) and up to a 3 month (Riou et al., 2019) exercise period have also demonstrated reductions in NEAT. Altogether, it is unclear how NEAT levels are affected by exercise. One interesting finding from our study was the negative correlation between GDF15 and total EE, showing that higher levels of GDF15 were associated with reduced EE. It has previously been observed in animal research that GDF15 lowers spontaneous activity (Klein et al., 2022). Although more research is required to elucidate the relationship between GDF15 and NEAT, the exercise‐induced increase in GDF15 may have an influence on postexercise activity levels.

This study is not without limitations. It is acknowledged that a liquid diet for 4 consecutive days does not represent a typical diet. Given that participants did not need to prepare or purchase meals, it is possible that the ease of consumption promoted an increase in EI. Alternatively, repeated consumption of an MRB may not be as palatable as normal food items and would thus drive down consumption. EI from the MRBs, however, was very similar to daily EE and intake remained consistent across the trials. In this way, despite the limitation of MRBs, the intake may better reflect homeostatic energy need than ad libitum meals and snack bags. Additionally, in order to measure free‐living EI, the timing of MRB consumption could not be standardized. This limitation prevented the assessment of appetite‐regulating hormones during the postprandial phase on day 3 and 4. Also, given that nutrient load can affect perceived appetite, individual consumption patterns may have influenced subjective measures of appetite at the specified recording times. Finally, the measurement of EE was assessed through indirect means using the activPAL. Although activity trackers have been demonstrated to be a valid and reliable tool (Grant et al., 2006), the tracker may not always accurately reflect EE and has been demonstrated to underestimate moderate‐vigorous physical activity (Wu et al., 2021, 2022). This limitation may have affected the energy balance measures.

In conclusion, this study sought to examine the impact of a 75 min session of moderate to vigorous exercise on appetite and energy compensation over a 3‐day postexercise window. To reduce some of the uncertainty around EI measurements in a free‐living environment, participants exclusively consumed MRBs. It was observed that exercise transiently modified appetite‐regulating hormones and elevated EE on the day of exercise, but no changes were observed in EI or EE on day 3 and 4 of each trial. Participants in the exercise condition reported a reduction in overall perceived satiety. Thus, a session of aerobic exercise appears to increase overall appetite while simultaneously enabling individuals to more effectively control intake in the days following an exercise session. Increases in energy compensation are unlikely to occur with an acute exercise session. Rather, energy compensation may gradually emerge after repeated exposure to exercise.

## FUNDING INFORMATION

This work was supported by a research grant through the Natural Sciences and Engineering Research Council (NSERC RGPIN‐2018‐05091).

## CONFLICT OF INTEREST STATEMENT

The authors declare no conflicts.

## ETHICS STATEMENT

The study was approved by the University of Lethbridge Human Participant Research Committee (Ethics ID #2020–058) and was conducted in accordance with the ethical principles of the *Declaration of Helsinki*.

## CONSENT

Informed consent was obtained from all participants involved in the study.

## Data Availability

The data will be made available upon request.
