# Correction to “Aerobic exercise elevates perceived appetite but does not modify energy intake over a 3‐day postexercise period: A pilot study”

**DOI:** 10.14814/phy2.70821

**Published:** 2026-03-17

Okada, T. E., Jeromson, S., Rathwell, S., Wright, D. C., & Bomhof, M. R. (2024). Aerobic exercise elevates perceived appetite but does not modify energy intake over a 3‐day postexercise period: A pilot study. *Physiological Reports*, 12, e70066. https://doi.org/10.14814/phy2.70066

The American Physiological Society and The Physiological Society (UK) are issuing a Society Note to include an Editor Disclosure Statement that was accidentally omitted from this published article in *Physiological Reports*. It should have been part of the Conflict of Interest statement.

Editor Disclosure Statement

David C. Wright, a co‐author on this article, was a Deputy Editor of the journal at the time the article was published. The Societies verify that David C. Wright was not involved in the handling of the manuscript and did not have access to information regarding the peer‐review process or final disposition of the article. An alternate editor oversaw the peer‐review and decision‐making processes.

